# What delivery-related factors affect postpartum recovery? A systematic review

**DOI:** 10.1016/j.xagr.2025.100496

**Published:** 2025-04-05

**Authors:** Zayël Z. Frijmersum, Eva Van der Meij, Petra C.A.M. Bakker, Ralph De Vries, Johannes R. Anema, Judith A.F. Huirne

**Affiliations:** a Department of Obstetrics and Gynecology, Amsterdam University Medical Center, Vrije Universiteit Amsterdam, Amsterdam, The Netherlands (Frijmersum and Huirne); b Amsterdam Reproduction and Development Research Institute, Amsterdam, The Netherlands (Frijmersum and Huirne); c Department of Obstetrics and Gynecology, Amsterdam University Medical Center, Amsterdam Medical Center, Amsterdam, The Netherlands (Frijmersum, Bakker, and Huirne); d Department of Obstetrics and Gynecology, Noordwest Ziekenhuisgroep, Alkmaar, The Netherlands (Meij); e Medical Library, Vrije Universiteit, Amsterdam, The Netherlands (De Vries); f Department of Public and Occupational Health, Amsterdam University Medical Center, Vrije Universiteit Amsterdam, Amsterdam, The Netherlands (Anema)

**Keywords:** cesarean delivery, childbirth, functional ability, mode of delivery, perineal laceration, postpartum, recovery

## Abstract

**OBJECTIVE:**

This study aimed to identify the delivery-related factors that affect postpartum recovery.

**DATA SOURCES:**

The PubMed, Embase, and Web of Science databases were searched until April 2024 using the following terms: “Childbirth,” “Caesarean section,” “Complications,” “Recovery,” and “Time Factors.” Studies in English or Dutch were considered for inclusion.

**STUDY ELIGIBILITY CRITERIA:**

All studies that included participants aged ≥18 years who delivered a live-born singleton and that evaluated the effect of delivery-related factors on recovery of health, ability, and activity in the postpartum period with a minimum follow-up period of 6 weeks were included.

**METHODS:**

Data from the included studies were extracted, and quality assessment was performed using the Newcastle-Ottawa Scale.

**RESULTS:**

A total of 38 articles were included. Of note, 5 different factors related to delivery that could affect recovery were identified as follows: mode of delivery, perineal lacerations, birth experience, parity, and neonatal factors. Articles could evaluate multiple affecting factors. Outcome measures were related to (genitopelvic or surgical site) pain, incontinence, mental health, and functional ability. Of note, 8 articles reported a negative effect on at least one of the outcome measures after cesarean delivery, 4 articles reported no significant difference between the delivery modes, and 2 articles found a negative effect on one of the outcome measures after vaginal delivery compared with cesarean delivery. Most articles (14/17) on perineal trauma reported a negative effect on recovery regarding incontinence and perineal pain. A negative birth experience was significantly associated with postpartum depression up to 6 weeks after childbirth. Parity of >2 was associated with more dyspareunia, and a high neonatal birthweight was associated with more pelvic pain.

**CONCLUSION:**

Our study findings indicate that mode of delivery, particularly cesarean delivery, is most frequently reported as having an effect on postpartum recovery. Recovery took longer (and was more painful) after cesarean delivery than after vaginal delivery. Extensive (third- and fourth-degree) perineal lacerations are frequently reported as an affecting factor. A small number of articles used functional ability as an outcome measure and attention for social participation.

**AJOG Global Reports at a Glance**

**Why was this study conducted?**

This study aimed to provide healthcare providers with a better insight into the recovery process of women after childbirth, which will lead to more personalized guidance in the recovery period to improve maternal health and social well-being.

**Key findings**

Mode of delivery was most frequently reported as having an effect on postpartum recovery. Functional ability as an outcome measure and attention for social participation was lacking.

**What does this add to what is known?**

Postpartum recovery is influenced by various delivery-related factors, such as mode of delivery.

## Introduction

The global number of childbirths in 2022 was approximately 133 million.^1^ This means that millions of women every year go through recovery after childbirth. However, this process remains poorly defined and inadequately explored. Postpartum recovery is a process that affects not only physical and mental health but also the social well-being of a mother.^2^ It is affected by many factors and processes, which can either improve or hinder recovery. Physical recovery during the postpartum period consists of 3 phases. The initial or acute phase involves the first 6 to 12 hours after delivery. The second phase is the subacute postpartum period, which lasts 6 weeks. The final phase is the delayed postpartum period, which can last up to 6 months.^3^ Recovery of social participation may take longer in terms of resumption of employment.^4^ Guidance during this recovery process is limited. The 6-week postpartum checkup is most commonly used in most countries to assess postpartum recovery and is the only contact with healthcare professionals after childbirth for most women, regardless of the course of their pregnancy, delivery, and recovery during their postpartum period.^5^

Defining and measuring recovery are difficult, as recovery can be considered a biomedical or biopsychosocial process.^6^ To get a better understanding of recovery as a process, it is important to understand the definition of health. Theoretical models of health outcomes or the consequences of disease have been models developed by the World Health Organization.^7^ The International Classification of Functioning, Disability and Health (ICF) is based on a biopsychosocial model that integrates medical and social models. The ICF identifies 3 main distinct components, impairment, activity limitation, and participation restriction, and their respective opposites, body function and structure, activity, and participation. Therefore, the concept of health can be defined as the ability to adapt and self-manage in the face of social, physical, and emotional challenges.^8^ Therefore, functional ability should be assessed in the domains of physical, mental, and social health as it is an outcome measure that relates to long-term health effects and societal participation.^9^^,^^10^ Literature shows that up to 90% of women deal with health-related problems during the first year after childbirth, such as extreme tiredness, sexual disorders, depression, and back pelvic and abdominal pain.^2^^,^^3^^,^^6^^,^^11^ Consequently, 30% of these women have not resumed employment after maternity leave.^12^^,^^13^ This indicates that there is an urgent need to prioritize postpartum recovery to improve women’s functional ability, as defined by the ICF.

Different personal, pregnancy, and delivery-related factors affect postpartum recovery.^14^^,^^15^ Here, we focused on delivery-related factors, as it has been shown that these factors are important predictors of prolonged sick leave.^16^ These factors relate to delivery from the start of the delivery process (such as mode of delivery) until after birth. Various studies have been conducted to evaluate the relationship between several delivery-related factors and how they affect postpartum recovery.^17^, ^18^, ^19^ However, most of these studies focused only on a single domain of recovery. No overview exists of all delivery-related factors and their role in the different domains of recovery, which is important as postpartum recovery is a complex process involving biomedical and psychosocial aspects.

## Objective

This systematic review aimed to identify the delivery-related factors that affect mental, physical, and functional recovery after childbirth. Based on the results, we aim to facilitate more personal guidance to mothers at risk of delayed recovery to improve maternal health and social participation and to prevent negative long-term health effects.

## Materials and methods

### Literature review

The conduct and reporting of this review were in accordance with the Preferred Reporting Items for Systematic Reviews and Meta-Analyses (PRISMA) guidelines^20^ and registered in the International Prospective Register of Systematic Reviews in advance.

### Eligibility criteria

The following inclusion criteria were used for selection.

#### Type of studies

We included cohort studies, both prospective and retrospective, and randomized controlled trials (RCTs). Studies must contain information about factors influencing postpartum recovery, with a minimum follow-up duration of at least 6 weeks after childbirth. Only studies written in Dutch or English were eligible for inclusion.

#### Type of participants

Women aged ≥18 years with a live-born child were included in the study.

#### Type of affecting factors

Studies evaluating the relationship between delivery-related factors and recovery after childbirth were included. A comparison was made regarding recovery between patient groups exposed to the affecting factor (affecting factor group) and patient groups not exposed to the affecting factor (comparison group).

Studies on personal and social factors were not included in this review. The findings will be presented in a second review in which we will focus more on biopsychosocial factors related to postpartum recovery.

#### Type of outcome measures

We included outcome measures for recovery in the postpartum period based on the concepts of health defined by the ICF, namely, components of body function and structure, activity, and participation.^21^ Therefore, outcome measures focusing on the physical, mental, and functional aspects of recovery were included. Outcomes regarding anatomic changes were not considered for inclusion in women who did not experience morbidity from these anatomic changes.

### Search strategy

To identify eligible publications, we conducted systematic searches in collaboration with a medical information specialist from inception to April 2024 in the following databases: PubMed, Embase, and Web of Science (core collection).

The following terms were used (including synonyms and closely related words) as index terms or free-text words: “Postpartum period,” “Recovery of Function,” “Obstetric Labour Complications,” and “Childbirth.” The references of identified articles were searched in relevant publications. We accepted articles written in Dutch and English. Duplicates were excluded using EndNote (version 20.0.1; Clarivate, Philadelphia, PA), following the Amsterdam Efficient Deduplication method^22^ and the Bramer method.^23^ The full search strategies for the used databases with the index terms can be found in Appendix.

### Study selection

Of note, 2 reviewers (Z.Z.F. and E.V.D.M.) independently screened all potentially relevant titles and abstracts for eligibility. If necessary, the full-text article was checked for eligibility criteria. A third reviewer (P.C.A.M.B.) was consulted in case of conflict between the first 2 reviewers regarding the inclusion or exclusion of articles. The remaining differences in judgment were resolved through a consensus procedure among the 3 reviewers.

### Data extraction

A data extraction form was developed. Data were extracted by 1 reviewer (Z.Z.F.) and were checked by the second reviewer (E.V.D.M.). Conflicts were discussed, and if necessary, a third reviewer (P.C.A.M.B) was consulted.

### Data items

From the included studies, we extracted data on (1) study characteristics (authors, year of publication, study design, type of affecting factors measured, number of participants included, and time of inclusion of participants), (2) study participant characteristics (inclusion and exclusion criteria, intervention, parity, and mean age), (3) type of affecting factors/interventions, (4) type of control group, and (5) outcome (type of outcome measure, methods of assessing outcome measure, timing of assessing outcome measure, and follow-up duration).

### Assessment of risk of bias

The assessment of quality for the nonrandomized studies was performed using the Newcastle-Ottawa Scale (NOS)^24^: form for cohort studies and cross-sectional studies. Of note, 1 reviewer (Z.Z.F.) independently assessed the quality of the included studies. The scoring system was used to assess the quality of the 3 broad perspectives: the selection of study groups, the comparability of the groups, and the ascertainment of either the exposure or outcome of interest. The quality categories defined using NOS are good, fair, and poor.

### Data synthesis

Because of the large heterogeneity in terms of the type and number of affecting factors, intervention, control groups, and type of outcome measures, it was not possible to conduct a meta-analysis. Therefore, we used narrative synthesis to describe and integrate the findings without formal statistical pooling.

## Results

### Search results

**Figure 1:**
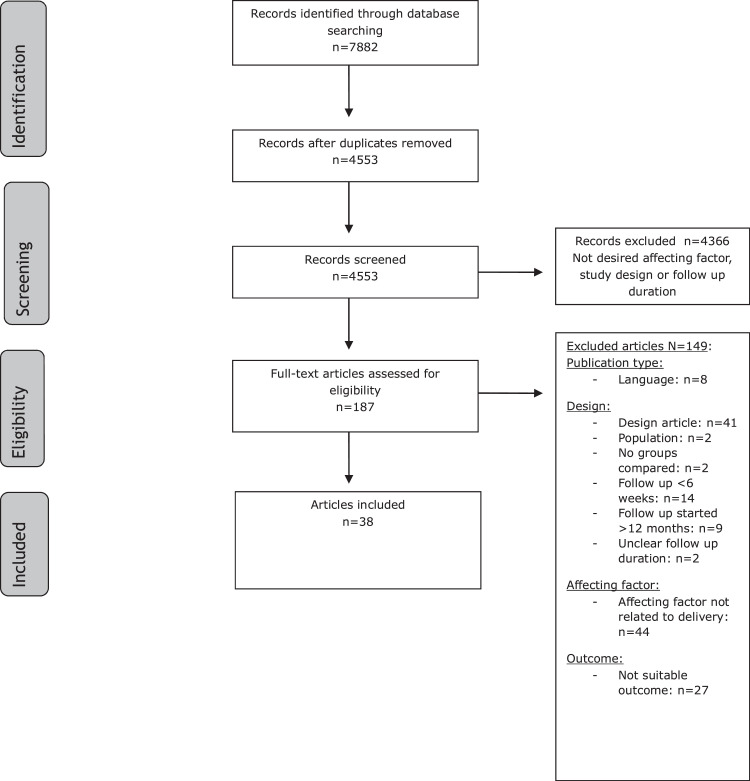
Flowchart of the search and selection procedure of studies

A total of 7882 references were identified for further evaluation: 2453 in PubMed, 3389 in Embase, and 2040 in Web of Science. After removing the duplicates, 4553 references remained. A flowchart of the search and selection processes is presented in Figure 1.

Frijmersum. Delivery related factors affecting postpartum recovery. Am J Obstet Gynecol Glob Rep 2025.

### Designs of the included articles

Of the 38 included studies (references in the Table^25^, ^26^, ^27^, ^28^, ^29^, ^30^, ^31^, ^32^, ^33^, ^34^, ^35^, ^36^, ^37^, ^38^, ^39^, ^40^, ^41^, ^42^, ^43^, ^44^, ^45^, ^46^, ^47^, ^48^, ^49^, ^50^, ^51^, ^52^, ^53^, ^54^, ^55^, ^56^, ^57^, ^58^, ^59^, ^60^, ^61^, ^62^), most (n=33) were cohort studies. Of the 38 studies, 25 were prospective cohort studies, and 8 were retrospective cohort studies. The remaining 5 studies consisted of 2 cross-sectional studies, 1 longitudinal study, 1 RCT, and 1 retrospective case-control study.

**Table 1:** Study characteristics

Study characteristics		Patients		Intervention group		Control group	Outcome			
Author name and year	Design	Parity	n	Type of affecting factor	Affecting factor category		Type of outcome measure	Follow-up duration	Quality score^a^	Result^b^
Tulman and Fawcett,^25^ 1988	Retrospective cohort	Primipara, multipara	70	CD	Mode of delivery	VD	Resumption of household, social and occupational activities, infant care	6 wk	Good	+; household tasks, societal participation X; employment resumption
Goffeng et al,^26^ 1998	Retrospective cohort	Primipara, multipara	42	OASI	Perineal lacerations	No OASI	Incontinence, dyspareunia	12 mo	Good	+; flatus incontinence, dyspareunia X; fecal and urinary incontinence
Kammerer et al,^27^ 1999	Prospective cohort	Primipara, multipara	30	OASI	Perineal lacerations	No OASI	Anal incontinence	4 mo	Good	+; more and worse anal incontinence
Goetsch et al,^28^ 1999	Prospective cohort	Primipara, multipara	62	Parity ≥3	Parity	Parity 1 or 2	Dyspareunia	12 mo	Good	−; less dyspareunia
Zetterström et al,^29^ 1999	Prospective cohort	Primipara	278	1. Assisted VD 2. OASI	1. Mode of delivery 2. Perineal lacerations	1. Spontaneous VD 2. No OASI	Anal incontinence	9 mo	Good	+; more anal incontinence
Abramowitz et al,^30^ 2000	Prospective cohort	Primipara, multipara	233	1. Assisted VD 2. OASI	1. Mode of delivery 2. Perineal lacerations	1. Spontaneous VD 2. No OASI	Anal incontinence	8 wk	Good	+; more anal incontinence
De Leeuw et al,^31^ 2001	Retrospective cohort	Primipara	250	OASI	Perineal lacerations	No OASI	Anal incontinence	14 y	Good	+; more anal incontinence X; urine incontinence
Fitzpatrick et al,^32^ 2002	Prospective randomized controlled trial	Primipara	178	Immediate pushing	Mode of delivery	Delayed (1 h)	1. Assisted delivery 2. Incontinence	6 mo	Good	X; no difference in assisted delivery or incontinence
Peschers et al,^33^ 2003	Retrospective cohort	Primipara	100	Assisted VD	Mode of delivery	Spontaneous VD	Incontinence	—	Poor	X; no difference in anal and urine incontinence
Nichols et al,^34^ 2005	Prospective cohort	Primipara	56	Fourth-degree perineal laceration	Perineal lacerations	Third-degree perineal lacerations	Anal incontinence	6 wk	Good	+; more incontinence
Jansen et al,^35^ 2007	Prospective cohort	Primipara, multipara	141	1. CD 2. Unplanned CD	Mode of delivery	1. VD 2. Elective CD	Health related quality of life	6 wk	Poor	+; higher fatigue scores in CD than in VD and in emergency CD than in elective CD
Eisenach et al,^36^ 2008	Prospective cohort	Primipara, multipara	939	CD	Mode of delivery	VD	Postpartum depression	8 wk	Good	X; no difference
Zaers et al,^37^ 2008	Prospective cohort	Primipara, multipara	47	Negative birth experience	Birth experience	Positive birth experience	Postpartum depression, PTSD	6 mo	Good	+; more depressive symptoms
Rogers et al,^38^ 2009	Prospective cohort	Primipara, multipara	326	Major perineal trauma	Perineal lacerations	Minor perineal trauma	Sexual function	3 mo	Good	X; no difference in activity at 3 months
Leeman et al,^39^ 2009	Prospective cohort	Primipara, multipara	343	Major perineal trauma	Perineal trauma	Minor perineal trauma	Dyspareunia	3 mo	Good	X; no difference in pain
Badiou et al,^40^ 2010	Retrospective cohort	Primipara	184	1. Assisted VD 2. OASI	1. Mode of delivery 2. Perineal lacerations	1. Spontaneous VD 2. No OASI	Anal incontinence	15 mo	Good	+; more anal incontinence
Kainu et al,^41^ 2010	Retrospective cohort	Primipara, multipara	438	CD	Mode of delivery	VD	Persistent pain	12 mo	Good	+; persistent pain
Marsh et al,^42^ 2011	Prospective cohort	Primipara, multipara	435	1. Assisted VD 2. Internal anal sphincter	1. Mode of delivery 2. Perineal lacerations	1. Spontaneous VD 2. External anal sphincter	Incontinence	3 mo	Good	+; more incontinence after assisted VD X; no difference in incontinence
Woolhouse et al,^43^ 2012	Prospective cohort	Primipara	1431	1. CD 2. Assisted VD	Mode of delivery	1. VD 2. Spontaneous VD	Incontinence, tiredness, back pain	18 mo	Good	1 +; more extreme tiredness, back/pelvic pain after CD 1 −; urine incontinence 2 +; incontinence after assisted VD
Hosseini et al,^44^ 2012	Retrospective cross-sectional	Primipara	213	CD	Mode of delivery	VD	Sexual function	24 mo	Fair	X; sexual function
Persico et al,^45^ 2013	Prospective cohort	Primipara, multipara	451	Perineal laceration or episiotomy	Perineal lacerations	No perineal laceration	Dyspareunia	6 mo	Good	+; delayed resumption sexual function X; dyspareunia
Liu et al,^46^ 2013	Prospective cohort	Primipara, multipara	426	Unplanned CD	Mode of delivery	Elective CD	Persistent pain	12 mo	Good	X; no difference in persistent pain
Bjelland et al,^47^ 2016	Longitudinal	Primipara, multipara	20.248	1. CD 2. Unplanned CD 3. Assisted VD 4. High fetal birthweight	1–3. Mode of delivery 4. Neonatal	1. VD 2. Elective CD 3. Spontaneous VD 4. Lower fetal birthweight	Pelvic pain	24 mo	Fair	1 −; pelvic pain 2 X; pelvic pain in unplanned and elective CD 3, 4 +; pelvic pain
Sayed Ahmed et al et al,^48^ 2017	Prospective cohort	Primipara, multipara	156	Major lacerations	Perineal lacerations	Minor perineal lacerations	Sexual function	12 mo	Good	+; decrease in FSF
Komatsu et al,^49^ 2017	Prospective cohort	Primipara	134	CD	Mode of delivery	VD	Self-assessed functional ability	3 mo	Good	+; functional recovery and pain resolution
Munro et al,^50^ 2017	Prospective cohort	Primipara	133	CD	Mode of delivery	VD	Persistent pain	3 mo	Good	X; no difference in persistent pain
Komatsu et al,^51^ 2018	Prospective cohort	Primipara	213	Induction of labor	Mode of delivery	Spontaneous onset of labor	Self-assessed functional recovery	4 mo	Fair	+
Anglès-Acedo et al,^52^ 2019	Prospective case-control	Primipara, multipara	318	OASI	Perineal lacerations	No OASI	Sexual function	6 mo	Good	+; worse sexual function
Molin et al,^53^ 2020	Prospective cohort	Primipara, multipara	1171	CD	Mode of delivery	VD	Persistent pain	8 mo	Good	+
Rosseland et al,^54^ 2020	Prospective cohort	Primipara, multipara	645	Negative birth experience	Birth experience	Normal birth experience	Postpartum depression	8 wk	Good	+; more depression
Gommesen et al,^55^ 2020	Prospective cohort	Primipara	575	OASI	Perineal lacerations	No OASI	Anal incontinence	12 mo	Good	+
Manresa et al,^56^ 2020	Prospective cohort	Primipara, multipara	405	Major perineal trauma	Perineal lacerations	Minor perineal lacerations	Perineal pain /dyspareunia	6 mo	Good	+; more perineal pain and dyspareunia
Sega et al,^57^ 2021	Retrospective cohort	—	213	Unplanned CD	Mode of delivery	Planned CD	Postpartum depression	12 mo	Good	+; higher incidence and severity
Attanasio et al,^58^ 2024	Prospective cohort	Primipara	2013	OASI	Perineal lacerations	No OASI	Anal incontinence	36 mo	Good	+; at 1 mo X; at 6 mo
Rosen et al,^59^ 2022	Prospective cohort	Primipara	582	Mode of delivery, extensive perineal lacerations	Mode of delivery, perineal lacerations	Mode of delivery, minimal perineal lacerations	Dyspareunia	24 mo	Good	X: trajectory of dyspareunia
Zhuang et al,^60^ 2023	Prospective cohort	Primipara, multipara	451	Unsuccessful trial of labor + nonlabor analgesia	Mode of delivery, analgesia	Unsuccessful trial of labor + labor analgesia	Postpartum depression	3 mo	Good	X: higher risk of postpartum depression at 3 mo
Chandra and Smitha,^61^ 2023	Cross-sectional	Primipara, multipara	220	CD	Mode of delivery	VD	Functional recovery	Unknown	Good	+; lower functional status at 6 wk after delivery
Xu and Sampson,^62^ 2023	Retrospective cohort	Unknown	2400	CD	Mode of delivery	VD	Pain	Unknown	Good	+; important predictor for postpartum pain

*CD*, cesarean delivery; *FSF*, Female Sexual Function; *OASI*, obstetrical anal sphincter injury; *PTSD*, posttraumatic stress disorder; *VD*, vaginal delivery.

a Newcastle-Ottawa Scale quality assessment score: good (5–9), fair (5–7), and poor (<5)

b The “+” symbol indicates significant effect in favor of the group exposed to the affecting factor (intervention), the “−” symbol indicates significant effect in favor of the nonexposed group (control), and the “X” symbol indicates no significant difference between the groups in terms of the affecting factor.

A total of 37 studies evaluated the relationship between specific delivery-related factors (affecting factors) and recovery. Of note, 1 study evaluated the effect of a specific intervention on recovery. Studies evaluating an affecting factor (A) made a comparison with a group that was not exposed to the affecting factor (B). For example, when the effect of induction of labor was evaluated on postpartum recovery, this was compared with a group of women in whom labor was not induced. The follow-up duration varied from a minimum of 6 weeks to 36 months after delivery.

### Participants

All participants had a mean age of 18 years (interquartile range, 18–50 years). Most studies (n=22) included both primiparous and multiparous participants. In 14 studies, only primiparous patients were included, and in 2 studies, parity was not mentioned.

### Type of affecting factors/interventions

We identified 5 different categories of delivery-related factors or interventions affecting postpartum recovery (affecting factors). These were factors or interventions focused on mode of delivery, perineal lacerations, birth experience, parity, and neonatal factors.

### Type of outcome measures

Different outcome measures were used. The most frequently used outcome measures were incontinence (n=13) and genitopelvic pain (n=13). The other categories were functional ability (n=5), mental health (n=5), and sexual function (n=4). Studies should report multiple outcome measures.

### Quality assessment of included studies

The quality of the included studies was assessed using the NOS. Of note, 34 studies were determined as having good quality, 3 studies were determined as having fair quality, and 1 study was determined to have poor quality. The Table^25^, ^26^, ^27^, ^28^, ^29^, ^30^, ^31^, ^32^, ^33^, ^34^, ^35^, ^36^, ^37^, ^38^, ^39^, ^40^, ^41^, ^42^, ^43^, ^44^, ^45^, ^46^, ^47^, ^48^, ^49^, ^50^, ^51^, ^52^, ^53^, ^54^, ^55^, ^56^, ^57^, ^58^, ^59^, ^60^, ^61^, ^62^ presents the outcomes of the included studies.

### Affecting factors

**Figure 2:**
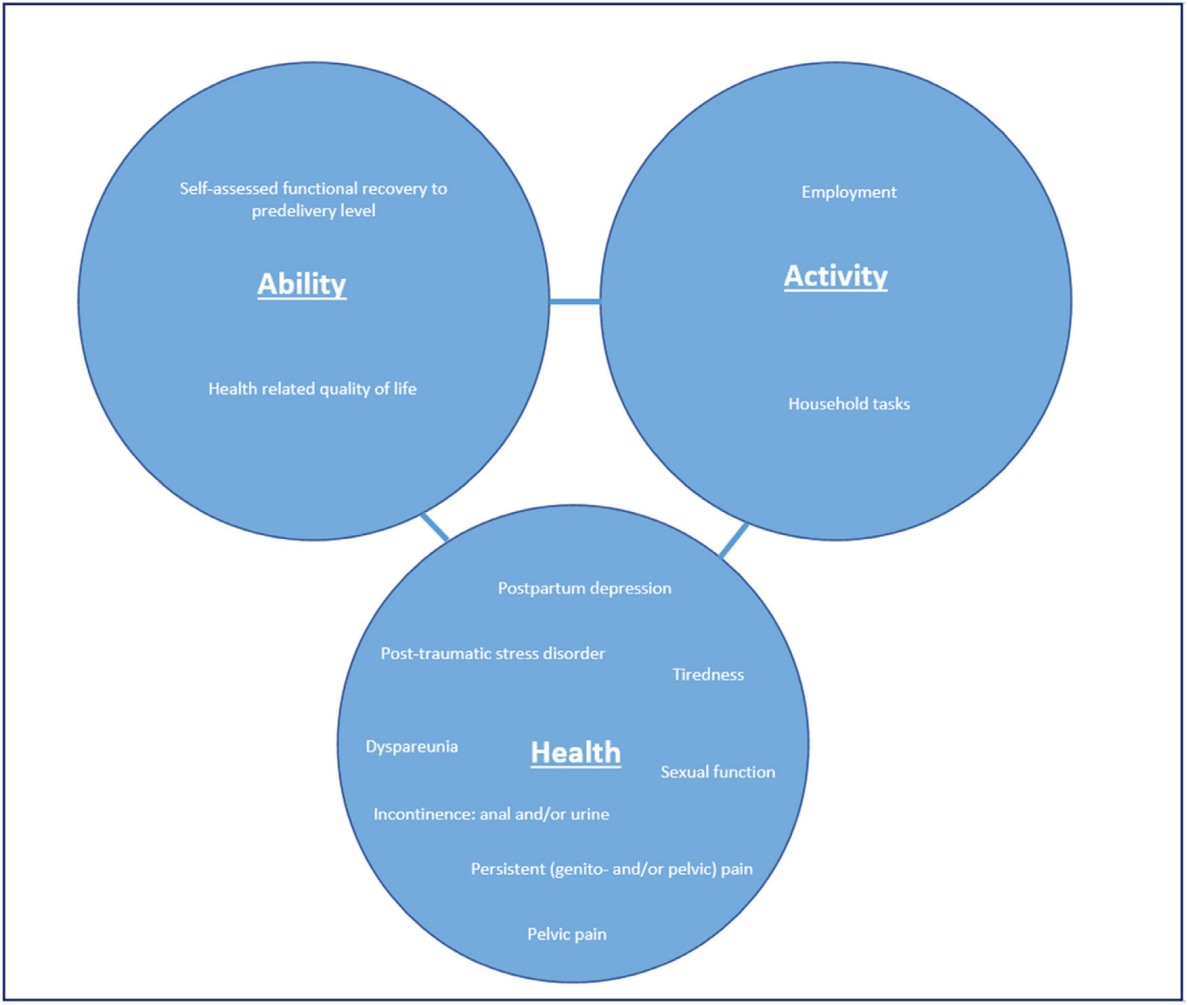
The concept of recovery The outcome measures for recovery are based on the concepts of health, ability, and activity according to the International Classification of Functioning, Disability and Health.

A total of 38 studies reported delivery-related factors that could affect postpartum recovery. Studies could report on multiple affecting factors affecting a certain outcome. Factors could be divided into 5 categories, namely, mode of delivery (n=23), type of perineal laceration (n=17), birth experience (n=2), parity (n=1), and neonate (n=1) (Figure 2).

Frijmersum. Delivery related factors affecting postpartum recovery. Am J Obstet Gynecol Glob Rep 2025.

## Mode of delivery

In 23 studies (60%), the influence of the mode of delivery on postpartum recovery was evaluated. Studies could report on multiple modes of delivery. In most studies (n=13), the effect of cesarean delivery (CD) vs vaginal delivery (VD) was described. In 7 studies, the effect of assisted VD vs spontaneous VD on recovery was evaluated. Of note, 4 studies reported on the influence of unplanned CD vs planned CD on recovery. Moreover, 1 study reported on the influence of induced delivery vs spontaneous delivery on postpartum recovery, 1 study reported on the influence of immediate pushing vs delayed pushing, and 1 study reported on the influence of nonlabor analgesia vs labor analgesia.

### Cesarean vs vaginal delivery

Women who had a CD needed significantly more time to return to functional ability than those with a VD regarding the resumption of household tasks, participation in professional and/or religious organizations, and socializing with friends (*P*<.001).^25^^,^^49^^,^^61^ In addition, significantly higher levels of fatigue scores were found after CD (mean ± standard error of the mean [SEM], 10.5±0.9) than after VD (mean±SEM, 9.9±0.9) at 6 weeks after delivery (*P*<.001).^35^ Resumption of occupational activities was not found to be significantly affected by the mode of delivery in a particular study.^25^ Of note, 1 study looked at the effect of mode of delivery on sexual function and found no significant differences in 6 domains concerning sexual function after CD compared with after VD at 2 years after delivery (*P*=.91).^44^

In addition, 2 studies reported that persistent pain (defined as pain at the surgical site or genital tract) 1 year after delivery was significantly more common after CD than after VD (*P*=.004 and *P*=.021, respectively).^41^^,^^53^ However, a different study did not support these findings. The studies observed no significant differences in chronic pain between the 2 modes of delivery at 3 months after delivery (*P*=.20).^50^ This finding was supported by a second study group that also found no significant relationship between mode of delivery and chronic pain (*P*=.34). CD was found to be an important predictor of pain after delivery compared with VD (permutation importance level of >0.08).^62^

Pelvic pain after delivery was more often observed in women after VD at 6 and 18 months after delivery than after CD (adjusted odds ratio [aOR], 0.48 [95% confidence interval (CI), 0.31–0.74]).^47^ In addition, no significant relationship was found between mode of delivery and postpartum depression (*P*=.56) 8 weeks after delivery.^36^ Of note, 1 study demonstrated more often back pain and extreme tiredness at 6 and 12 months after delivery in women after CD than in women after VD (aORs: 1.37 and 1.40, respectively).^43^ In addition, this study reported that the CD group was less likely to suffer from urine incontinence at 3, 6, and 12 months after delivery than the VD group (aORs: 0.26, 0.36, and 0.48, respectively).

### Induced vs spontaneous labor

In addition, 1 study reported on the effect of spontaneous and induced labor on recovery, defined as self-assessed functional recovery to predelivery level. The study reported a significant delay in return to functional ability in the induced labor group up to 4 months after delivery compared with the spontaneous onset of the labor group (*P*=.004).^51^

### Assisted vs spontaneous vaginal delivery

In addition, 4 studies reported that assisted VD was significantly associated with more anal incontinence complaints up to 15 months after delivery than spontaneous VD (*P*<.05).^29^^,^^30^^,^^40^^,^^42^ In contrast, 1 study found no difference in urinary or anal incontinence between assisted VD and spontaneous VD at 6 months after delivery.^33^ In addition, pelvic pain after delivery was reported in association with assisted VD by 1 study compared with spontaneous VD (odds ratio [OR], 1.30 [95% CI, 1.06–1.59]).^47^ It was reported that, after assisted VD, women resumed coitus significantly later and had worse sexual function than women who had a spontaneous VD (*P*<.001).^52^

### Immediate vs delayed pushing in vaginal delivery

Of note, 1 particular study evaluated the effect of immediate vs delayed (1 hour) pushing and found no significant difference in anal incontinence up to 3 months after delivery (*P*=.27).^32^

### Unplanned vs planned caesarean delivery

The time to full recovery was more than 6 weeks after unplanned CD than after planned CD (*P*<.001). However, it is not reported how long exactly*.*^35^ Unplanned CD was significantly associated with more frequent and severe postpartum depressive symptoms than planned CD (*P*=.038).^57^ No significant association was found in persistent pelvic pain or surgical site pain between the 2 groups.

### Nonlabor analgesia vs labor analgesia

Of note, 1 study looked at the effect of nonlabor analgesia vs labor analgesia during an unsuccessful trial of labor (resulting in CD) on postpartum depression at 3 months. They found that unsuccessful trial of labor while not receiving epidural analgesia did not have a higher risk of postpartum depression (OR, 0.331 [95% CI, 0.121–0.902]; *P*=.026).

## Perineal lacerations

A total of 17 studies reported that perineal lacerations affected postpartum recovery. The outcome mostly affected by perineal lacerations was anal incontinence*.*^26^^,^^27^^,^^29^^,^^30^^,^^31^^,^^34^^,^^40^^,^^55^^,^^58^ Anal incontinence was significantly more frequent and persisted longer in cases of obstetrical anal sphincter injury (OASI) than no OASI (*P*=.08^27^; OR, 3.0^29^; *P*=.004^30^; *P*=.004,^31^ OR, 18.7^34^; *P*=.02^40^; adjusted relative risk, 2.46^55^). In addition, 1 study reported more accidental stool loss in patients with OASI at 1 month after delivery than in those without OASI. However, at 6 months after delivery, there was no statistically significant difference in outcomes by OASI.^58^ A different study found no significant difference in urine incontinence and fecal incontinence at 6 weeks after delivery between patients with OASI and those without OASI.^26^ However, they did report significantly more involuntary flatus and dyspareunia in the OASI group (*P*<.01). A second study supports the finding of no difference in urinary incontinence between patients with OASI and those without OASI (OR, 1.46).^31^ In addition, 1 study found no difference in fecal symptoms between patients who suffered internal anal sphincter injuries and patients who suffered external anal sphincter injuries.^42^

The other outcomes affected by perineal lacerations were dyspareunia and sexual function. Of note, 2 studies reported significant lower female sexual function scores at 6 and 12 months after delivery in women who sustained major perineal trauma (third- and fourth-degree lacerations) than minor perineal trauma (first- and second-degree laceration) (*P*<.05^48^ and *P*<.001,^52^ respectively). In contrast, 3 other studies reported no overall differences in sexual activity and function at 3 months after delivery (*P*=.41,^38^ *P*=.34,^45^ and *P*=.92^59^).

Higher perineal pain scores and more analgesic use were observed with major perineal trauma up to 3 months after delivery (*P*=.003^45^; OR, 5.5 [95% CI, 2.8–10.9]^56^). Afterward, no difference in pain scores was observed between major trauma and minor perineal trauma (*P*=.77).^39^

## Negative birth experience

Of note, 2 studies reported on the effect of birth experience on postpartum recovery. Both studies reported that a negative birth experience was significantly associated with postpartum depression up to 6 weeks after childbirth ((*P*=0.038^37^ and *P*=.01^54^). No relationship was found between birth experience and persistent genitopelvic pain up to 8 weeks after delivery (*P*=.73).^54^

## Parity

Only 1 study evaluated the effect of parity on postpartum recovery. This study found that women who delivered their first or second child experienced dyspareunia more often than multiparous women.^28^

## Neonatal factors

In addition, 1 study reported on the influence of neonatal birthweight on recovery after delivery. A high neonatal birthweight was found to be associated with new-onset pelvic pain.^47^ The odds of reporting pelvic pain increased by 34% (aOR, 1.34 [95% CI, 1.17–1.53]) for every increase in neonatal birthweight by 1 kg. However, the relationship between the mode of delivery and high fetal birthweight concerning pelvic pain was not described.

## Discussion

### Principal findings

In this systematic review, we evaluated which delivery-related factors affect postpartum recovery, which is defined as maternal recovery after childbirth. Recovery was defined by concepts of health, ability, and activity related to the ICF. A total of 38 studies were included in this study. The evaluated studies were heterogeneous regarding number of patients, type of affecting factors, interventions, outcome measures, and follow-up duration.

The most frequently reported outcome measures were the concept of physical health conditions, such as incontinence (n=13 [34%]) and genitopelvic or surgical site pain (n=12 [31%]). Mental health was used as an outcome measure in 5 studies (13%). The concept of functional ability after delivery was evaluated in only 5 studies (13%), including 2 studies that defined functional ability as self-assessed functional recovery to predelivery level.

Mode of delivery was the affecting factor that was most evaluated (n=23 [60%]). Of note, 12 studies evaluated the effect of CD vs VD on postpartum recovery. Moreover, 9 studies (75%) reported a negative effect of CD on recovery compared with VD, 4 studies (33%) reported no significant difference between the delivery modes, and 2 studies (16%) found a negative effect on recovery after VD compared with after CD.

In addition, 7 studies evaluated the effect of assisted VD vs spontaneous VD. Moreover, 6 studies (86%) found a negative effect on recovery after assisted VD. However, 1 study (14%) found no difference between both groups. Furthermore, 4 studies compared the difference between unplanned CD and planned CD on recovery. In 2 studies (50%), a negative effect on recovery was reported after unplanned CD. However, in the other 2 studies, no effect was found between both groups. Induction of labor was reported to delay self-assessed functional recovery compared with spontaneous onset of delivery. An unsuccessful trial of labor without epidural analgesia was not found to have a higher risk of postpartum depression.

Of note, 17 studies evaluated the effect of perineal lacerations on recovery using multiple outcome measures.

In addition, 14 studies (82%) reported that major perineal trauma (third- or fourth-degree laceration) or OASI had a negative effect on recovery compared with minor perineal trauma (no or first-/second-degree laceration) or no OASI. Moreover, 5 studies (31%) reported no significant difference in recovery between the groups. Finally, negative birth experience, first or second parity, and high neonatal birthweight were reported as factors that negatively influenced recovery.

### Comparison with existing literature

Only a few systematic reviews have been conducted that assessed the influence of 1 single delivery-related factor on a specific domain of postpartum recovery. Our findings are in accordance with these results. Manresa et al^18^ reported a higher prevalence of dyspareunia at 6 and 12 months after delivery in women with major perineal trauma after spontaneous VD. However, their review includes studies on different repair methods that we did not examine. Komatsu et al^19^ reviewed factors associated with persistent pain after childbirth. Similar to our findings, the authors found that perineal and vaginal pain was associated with tissue trauma related to assisted VD. Compared with spontaneous VD, assisted VD was consistently reported as a risk factor for persistent perineal pain. A survey study by Declercq et al^63^ reported no difference in persistent (perineal or surgical site) pain between women after elective CD and women after emergency CD. In addition, their findings support the findings of a higher prevalence of persistent perineal pain after assisted VD compared with spontaneous VD. However, their sample size was relatively small (n=36). Concerning the conduct of daily activities in the first 2 months after delivery, the authors found that women who underwent CD were more likely to report interference of pain with daily activities than women who underwent VD.

In systematic reviews by Pretlove et al^17^ and Cattani et al,^64^ significantly more women suffered from anal incontinence after assisted VD than spontaneous VD. The result was similar to our findings.^29^^,^^30^^,^^40^^,^^42^^,^^47^^,^^52^ Finally, reviews by Cattani et al^64^ and Bols et al^65^ observed a significant influence of OASI on anal incontinence.

### Interpretation of the results

Of note, 5 studies used functional ability as an outcome measure, reporting on resumption of social activities and self-assessed functional recovery to predelivery level.^25^^,^^35^^,^^49^^,^^51^^,^^61^ Tulman and Fawcett^25^ reported that CD had a negative effect on resumption of household tasks and societal participation compared with VD, but not on employment resumption. A possible explanation could be that the length of maternity leave was equal for both groups. Unfortunately, this was not disclosed in the study. Komatsu et al^49^^,^^51^ reported that CD and induction of labor delayed self-assessed functional recovery compared with spontaneous VD. Jansen et al^35^ and Chandra and Smitha^61^ reported lower health-related quality of life scores in patients who underwent CD than in those who underwent VD, defining health-related quality of life in the domains of physical, emotional, and social activities. The outcome measures used by these 5 studies illustrate that postpartum recovery is rarely assessed by functional ability. This is remarkable as several studies have revealed that the effect of childbirth on participation in society is significant. A study by Beukering et al^14^ showed that 30% of women are still absent from work after maternity leave. As pregnant women belong to a patient demographic with an important role in society, the effect of these effects is considerable. This illustrates that participation concerning postpartum recovery is underexposed in current literature.

Other studies reported negative effects of CD on recovery compared with VD, including physical symptoms, such as fatigue^35^^,^^43^ and persistent pain (defined as surgical site or genitopelvic pain).^41^^,^^53^^,^^62^ Kainu et al^41^ and Molin et al^53^ reported CD as an affecting factor for persistent pain compared with VD, whereas Eisenach et al^36^ and Munro et al^50^ did not report this finding. The reason for this contradictory finding could be the larger population size and longer follow-up duration in the studies by Kainu et al^41^ and Molin et al.^53^ Both Eisenach et al^36^ and Kainu et al^41^ adjusted persistent pain for acute pain after delivery with contrasting results, but only Kainu et al^41^ adjusted for patient history of chronic disease and previous pain. Considering the worldwide increasing CD rates, effects on maternal health should be considered, as they are very likely to influence maternal functional ability, including societal participation.^66^

Assisted VD has been identified as a significant factor influencing postpartum recovery, particularly concerning anal incontinence, pelvic pain, and diminished sexual function, compared with spontaneous delivery, likely due to the association with more extensive perineal lacerations.^29^^,^^30^^,^^40^^,^^42^^,^^47^^,^^52^ Therefore, the indication for assisted VD should be carefully evaluated.

Similarly, induction of labor has been reported to affect recovery, specifically regarding self-assessed functional recovery to predelivery levels, although the small study sample sizes necessitate cautious interpretation of these findings.^51^ In addition, labor analgesia for unsuccessful vaginal childbirth did not reduce the risk of postpartum depression compared with those who did not receive epidural analgesia. This may be due to the higher degree of cervical dilation and prolonged pain exposure before emergency CD in the labor analgesia group, indicating that the potential benefits of early labor pain analgesia warrant further investigation.^60^

Unplanned CD was found to have a negative effect on recovery compared with planned CD in terms of time to full recovery and postpartum depressive symptoms.^35^^,^^57^ Jansen et al^35^ reported that women who underwent an unplanned CD needed more than 6 weeks to reach full recovery (defined as mean scores of physical health-related quality of life scales, physical functioning and role physical) compared with women who underwent planned CD (6 weeks) and VD (3 weeks). This could be explained by the invasiveness of the different delivery methods. Patients who underwent an unplanned CD experienced both labor and an operative procedure, which may explain the worse health-related quality of life scores. In addition, the emotional aspects of an unplanned CD, such as symptoms of anxiety and depression, have been reported more often after an unplanned CD, resulting in affected participation in social activities and functioning.^57^ From this perspective, healthcare providers should take this into account when guiding patients through the recovery process after an unplanned CD. Interestingly, 2 other studies found no difference in chronic pain (surgical site or pelvic pain) between unplanned and planned CDs, suggesting that unplanned CD mainly affects psychosocial aspects of recovery and not so much biomedical aspects.^46^^,^^47^

Women who suffered extensive perineal trauma suffered significantly more from anal incontinence.^27^^,^^29^^,^^30^^,^^31^^,^^34^^,^^40^^,^^55^^,^^58^ Of note, 5 studies observed more dyspareunia and delayed resumption of sexual function after major perineal trauma up to 12 months after delivery.^26^^,^^45^^,^^52^^,^^48^^,^^56^ However, 3 studies did not support these findings at 3 months after delivery.^38^^,^^45^^,^^59^ No difference in follow-up duration or population size was found between these studies to possibly explain this discrepancy. Nevertheless, the effect of extensive perineal trauma on female sexual function should not be underestimated or overlooked and should be an incentive for immediate referral to specialized physical health therapists for treatment. Dyspareunia was significantly observed more often after a first or second VD, but not after more than 3 deliveries.^29^ A possible explanation could be the frequency of perineal trauma that is more often experienced by primiparous women. High neonatal birthweight was associated with a higher risk of pelvic pain than low neonatal birthweight.^47^ A higher birthweight might mean more mechanical pressure on the pelvis during pregnancy and delivery, resulting in pelvic pain.

Women with a negative birth experience seem to be more likely to suffer from postpartum depression. Therefore, attention to mental health should be recommended for this group of patients to prevent further health problems affecting social activities and participation.^37^^,^^54^

### Strengths and limitations

To the best of our knowledge, this systematic review provides an overview of all delivery-related factors affecting postpartum recovery. Postpartum recovery is a complex process that has an effect on outcome measures regarding all aspects of recovery. We have provided an overview of delivery-related factors affecting the different domains of recovery, including physical, mental, and functional recovery. Functional ability is defined as the ability to adapt and self-manage physical, emotional, and social activities.^10^ Considering most articles reported on the physical domain of recovery, this review is unique in the approach and assessment of postpartum recovery in light of all domains of recovery (physical, mental, and functional). Another quality of this review is the methodologic approach following the PRISMA guidelines for systematic reviews.^20^ A very broad literature search with wide inclusion criteria was performed in collaboration with a medical information specialist. Therefore, we were able to include an extensive number of studies to provide a broad overview of delivery-related affecting factors influencing postpartum recovery.

A possible limitation could be the decision to only include delivery-related affecting factors and not personal and pregnancy-related factors. Because a large number of studies yielded after the first literature search, the decision was made to limit inclusions to delivery-related factors. A broader scope of biopsychosocial factors, including personal and external factors, will be evaluated in a second review. By only including delivery-related factors in this review, there could have been bias toward certain specific factors, such as the mode of delivery. This could result in the underestimation of existing literature, which mentions other different factors. By using broad search terms, we have tried to limit this bias. A second possible limitation could be that outcomes regarding anatomic changes were not considered for inclusion in women who did not experience morbidity from these anatomic changes. This could result in an under- or overreporting of affecting factors. Finally, it was not possible to conduct a meta-analysis because of heterogeneity regarding the type of affecting factors, outcome measures, and follow-up duration. This diversity reflects the context of childbirth. Narrative synthesis supports this complexity, providing a strong foundation for clinical guidance and future research into uniform recovery definitions.

### Implications for research and practice

Given the increasing number of CD and assisted VD worldwide, physicians should be more aware of the effect that mode of delivery and the extent that perineal trauma have on physical recovery after childbirth, as this affects maternal functioning.^67^ Our focus on ICF domains of recovery reveals the underexposure of participation (13% of studies), despite its significant societal effect. For example, 30% of individuals do not return to work after childbirth.^12^^,^^13^

Future studies should use functional ability to measure recovery as it encompasses physical, mental, and social components of health, and current literature shows that it is underexposed, yet the societal consequences are significant. In the second review, we will focus on personal and pregnancy-related factors affecting different biopsychosocial components regarding postpartum recovery. By gaining a better understanding of which factors affect postpartum recovery, clinicians are able to provide patients with more information and personalized healthcare.

## Conclusion

Our review highlights that postpartum recovery is generally viewed as recovery of health concept, such as biomedical symptoms and complications, as opposed to recovery viewed as an ability concept or as an activity concept. Our results showed that postpartum recovery is influenced by various delivery-related factors, such as the mode of delivery and the extent of perineal lacerations, which increase the risk of long-term health complaints.

## CRediT authorship contribution statement

**Zayël Z. Frijmersum:** Writing – review & editing, Writing – original draft, Methodology, Formal analysis, Data curation, Conceptualization. **Eva Van der Meij:** Writing – review & editing, Supervision, Data curation, Conceptualization. **Petra C.A.M. Bakker:** Writing – review & editing, Supervision. **Ralph De Vries:** Methodology, Data curation. **Johannes R. Anema:** Writing – review & editing, Supervision. **Judith A.F. Huirne:** Writing – review & editing, Conceptualization.

## Appendix Supplementary materials

Image, application 1
