# Supplementary material for: What delivery-related factors affect postpartum recovery? A systematic review
Source: AJOG Glob Rep. 2025 Apr 5;5(2):100496. doi: 10.1016/j.xagr.2025.100496 (PMC12127658; doi:10.1016/j.xagr.2025.100496)
Supplement: Supplementary file 1 [file mmc1.docx]

**Appendix A: Full search strategies for all databases**

**PubMed Results (10 Apr 2024)**

| Search | Query | Items found |
| --- | --- | --- |
| #5 | **#1 AND #2 AND #3 AND #4** | 2,453 |
| #4 | **"Time Factors"[Mesh] OR "time"[tiab] OR "period"[tiab] OR "periods"[tiab] OR "Postpartum Period"[Mesh:NoExp] OR "postdeliver*"[tiab] OR "post-deliver*"[tiab] OR "postpartum"[tiab] OR "post-partum"[tiab] OR "puerp*"[tiab] OR "after deliver*"[tiab]** | 6,164,417 |
| #3 | **"Enhanced Recovery After Surgery"[Mesh] OR "Recovery of Function"[Mesh] OR "Convalescence"[Mesh] OR "recover*"[tiab] OR "convalescen*"[tiab] OR "repair*"[tiab] OR "persistent pain"[tiab] OR "chronic pain"[tiab]** | 1,355,620 |
| #2 | **"Pregnancy Complications"[Mesh:NoExp] OR "Obstetric Labor Complications"[Mesh] OR "Puerperal Disorders"[Mesh] OR "pregnancy complication*"[tiab] OR "labor complication*"[tiab] OR "labour complication*"[tiab] OR "tear"[tiab] OR "tears"[tiab] OR "lacerat*"[tiab] OR "haemorrhage*"[tiab] OR "hemorrhage*"[tiab] OR "excessive bleeding*"[tiab] OR "analges*"[tiab] OR "pain"[tiab] OR "puerp*"[tiab] OR "hindrance*"[tiab]** | 1,384,069 |
| #1 | **"Delivery, Obstetric"[Mesh] OR "Parturition"[Mesh] OR "parturition"[tiab] OR "parturient*"[tiab] OR "birth*"[tiab] OR "childbirth*"[tiab] OR "vaginal deliver*"[tiab] OR "normal deliver*"[tiab] OR "forceps deliver*"[tiab] OR "cesarea*"[tiab] OR "caesarea*"[tiab] OR "c section"[tiab] OR "c sections"[tiab] OR "postcesarea*"[tiab] OR "postcaesarea*"[tiab] OR "obstetrical extraction*"[tiab] OR "vacuum extraction*"[tiab] OR "ventouse"[tiab] OR "episiotom*"[tiab] OR "fetal version*"[tiab] OR "foetal version*"[tiab]** | 544,589 |

**Embase.com Results (10 Apr 2024)**

| Search | Query | Items found |
| --- | --- | --- |
| #6 | **#5 NOT ('conference abstract'/it OR 'conference review'/it)** | 3,389 |
| #5 | **#1 AND #2 AND #3 AND #4** | 5,295 |
| #4 | **'time factor'/exp OR 'puerperium'/exp OR 'time':ab,ti,kw OR 'period':ab,ti,kw OR 'periods':ab,ti,kw OR 'postdeliver*':ab,ti,kw OR 'post-deliver*':ab,ti,kw OR 'postpartum':ab,ti,kw OR 'post-partum':ab,ti,kw OR 'puerp*':ab,ti,kw OR 'after deliver*':ab,ti,kw** | 7,384,308 |
| #3 | **'enhanced recovery after surgery'/exp OR 'convalescence'/exp OR 'recover*':ab,ti,kw OR 'convalescen*':ab,ti,kw OR 'repair*':ab,ti,kw OR 'persistent pain':ab,ti,kw OR 'chronic pain':ab,ti,kw** | 1,753,094 |
| #2 | **'pregnancy complication'/de OR 'labor complication'/exp OR 'puerperal disorder'/exp OR 'pregnancy complication*':ab,ti,kw OR 'labor complication*':ab,ti,kw OR 'labour complication*':ab,ti,kw OR 'tear':ab,ti,kw OR 'tears':ab,ti,kw OR 'lacerat*':ab,ti,kw OR 'haemorrhage*':ab,ti,kw OR 'hemorrhage*':ab,ti,kw OR 'excessive bleeding*':ab,ti,kw OR 'analges*':ab,ti,kw OR 'pain':ab,ti,kw OR 'puerp*':ab,ti,kw OR 'hindrance*':ab,ti,kw** | 2,099,158 |
| #1 | **'obstetric delivery'/exp OR 'birth'/exp OR 'episiotomy'/exp OR 'parturition':ab,ti,kw OR 'parturient*':ab,ti,kw OR 'birth*':ab,ti,kw OR 'childbirth*':ab,ti,kw OR 'vaginal deliver*':ab,ti,kw OR 'normal deliver*':ab,ti,kw OR 'forceps deliver*':ab,ti,kw OR 'cesarea*':ab,ti,kw OR 'caesarea*':ab,ti,kw OR 'c section':ab,ti,kw OR 'c sections':ab,ti,kw OR 'postcesarea*':ab,ti,kw OR 'postcaesarea*':ab,ti,kw OR 'obstetrical extraction*':ab,ti,kw OR 'vacuum extraction*':ab,ti,kw OR 'ventouse':ab,ti,kw OR 'episiotom*':ab,ti,kw OR 'fetal version*':ab,ti,kw OR 'foetal version*':ab,ti,kw** | 784,638 |

**Web of Science (Core Collection) Results (10 Apr 2024)**

| Search | Query | Items found |
| --- | --- | --- |
| #5 | **#1 AND #2 AND #3 AND #4** | 2,040 |
| #4 | **TS=("time" OR "period" OR "periods" OR "postdeliver*" OR "post-deliver*" OR "postpartum" OR "post-partum" OR "puerp*" OR "after deliver*")** | 9,667,942 |
| #3 | **TS=("recover*" OR "convalescen*" OR "repair*" OR "persistent pain" OR "chronic pain")** | 2,043,452 |
| #2 | **TS=("pregnancy complication*" OR "labor complication*" OR "labour complication*" OR "tear" OR "tears" OR "lacerat*" OR "haemorrhage*" OR "hemorrhage*" OR "excessive bleeding*" OR "analges*" OR "pain" OR "puerp*" OR "hindrance*")** | 1,295,501 |
| #1 | **TS=("parturition" OR "parturient*" OR "birth*" OR "childbirth*" OR "vaginal deliver*" OR "normal deliver*" OR "forceps deliver*" OR "cesarea*" OR "caesarea*" OR "c section" OR "c sections" OR "postcesarea*" OR "postcaesarea*" OR "obstetrical extraction*" OR "vacuum extraction*" OR "ventouse" OR "episiotom*" OR "fetal version*" OR "foetal version*")** | 603,947 |
